# Examining students’ music listening willingness and engagement to foster their musical achievement and development in higher educational institutions

**DOI:** 10.1038/s41598-024-52911-w

**Published:** 2024-02-06

**Authors:** Xiaokang Wang, Wenrong Huang

**Affiliations:** Present Address: College of Music and Dance, Guizhou Minzu University, Guiyang, Guizhou China

**Keywords:** Psychology, Human behaviour, Auditory system, Cognitive neuroscience, Emotion, Learning and memory, Motivation, Reward, Social behaviour, Social neuroscience

## Abstract

Drawing upon self-determination theory, this study explores how listening music willingness (LMW) and music engagement (ME) impact musical development and achievement (MDA) via the mediating role of music aesthetic experience (MAE) and music listening behavior (MLB) among 299 university music students in Chinese higher educational institutions (HEIs). Employing structural equation modeling (SEM), the results reveal that LMW significantly influences MLB but not musical development and achievement. ME significantly affects both music aesthetic experience and MDA, with a significant correlation between MLB and MDA. Besides, the relationship between MAE and MDA is positively significant. Mediation analysis reveals that music listening behavior fully mediates the LMW–MDA relationship, while the relationship between ME and MDA is partially mediated by musical aesthetic experience. These findings offer insights for crafting music educational strategies emphasizing positive listening behavior, active engagement, and enriched aesthetic experiences to enhance effectiveness in HEIs. For policymakers particularly in China, understanding the pivotal role of MLB as a mediator between willingness and achievement suggests interventions targeting listening habits can positively influence overall MDA. Furthermore, recognizing the partial mediation by aesthetic experience in the relationship between engagement and MDA suggests programs enhancing musical aesthetic experiences could amplify the impact of music education initiatives.

## Introduction

### Significance of music education

Over the last few decades, development and achievement have peaked in every field in this highly competitive world, as development comprises progressive changes in abilities across the lifespan^1^. Likewise, educational development^2^, particularly musical education and its development, has become more advanced in recent decades^3^. Music education has a large influence and significance, particularly in Asian countries such as China^4^, where it has been practiced since ancient China. Xu^5^ believes that music education is the same as ethical and moral education and that music is a symbol of human goodness. Furthermore, educational institutions have benefited from adopting musical activities, which have increased the psychological satisfaction of students^6^. Additionally, music listening patterns and engagement seem to be able to address mental issues^7^. Therefore, musical engagement and listening behavior are becoming more critical to musical development.

Despite the scenario mentioned above, it is evident that musical development and achievement nowadays represent a challenging phenomenon that has radically transformed the way in the context of educational settings^8^, as well as the better understanding of the role that music plays in the lives of university students and the potential benefits that can be gained from engaging with music^9^. While extensive research has explored the association between motivational^10^, environmental^11^, and cognitive factors^12,13^ and musical development and achievement^14^. In current musical development and achievement literature, there needs to be more empirical evidence regarding the factors and their role in impacting musical development and achievement, particularly among university music students. Hence, the authors identified three gaps in the musical development and achievement-associated literature through a comprehensive review of prior studies, as indicated in Table 1.

**Table 1:** Overview of existing studies related to musical engagement and musical achievement and development.

Studies	Independent variables	Mediating variables	Dependent variables	Moderating variables	Country and sector contexts
^6^	Perceived teachers need and support	Needs satisfaction	Musical achievement and elective intentions	None	Australia
^15^	Academic self-efficacy and academic self motivation	Learning agility	Sustainable students engagement and academic achievement	None	Music students from Chinese institutions
^16^	Emotional, cognitive and background use of music	Cognitive reappraisal, and expressive suppression	Flourish in university students	None	Pakistani public universities
^17^	Interactive music	Perceived control, and vividness	User engagement	None	American university students
^18^	Music preferences and music use	Personality traits and the dark traid	Innovation and employees outcomes	None	Canadian students
^19^	Self theories of intelligence and musicality	Personality dimension and academic effort	Academic achievement	None	UK secondary school students
^12^	Music use	Immersion and aesthetic experience	e-behavioral intentions	Emotions and contemplation	French teenagers
^20^	Music emotional awareness	Positive emotions and negative emotions	Wellbeing	None	Hong kong secondary school students
^21^	Autonomous motivation	Flow-fluency of performance and absorption	Life satisfaction, positive and negative effects	None	German University musician
^8^	Intervention in music and humanities	General self-efficacy	Grades and qualification	None	Spanish school students

### Identified research gaps

First, numerous studies have shown that listening to music enhances cognitive abilities such as memory, attention, and problem-solving skills^8,13,22^. Listening to music and its effect on students' academic performance is an emerging topic and received enough attention recently^14,20,23^. Listening-music willingness refers to an individual's positive attitude and eagerness to engage in the activity of listening to music^18,24^. It signifies the level of interest, motivation, and openness someone has toward listening to music. Moreover, listening to active music can improve students' perception and interpretation of music^8^. It allows them to analyze musical elements such as melody, harmony, rhythm, and dynamics, enhancing their musical understanding and performance abilities^14^. University music students who willingly engage in music listening may experience enhanced cognitive abilities, leading to improved musical achievement and development^8,25^. Listening to music can inspire creativity and foster innovative thinking among university music students. It exposes them to various artistic approaches, techniques, and musical ideas that can fuel their creative endeavors. Increased willingness to engage in music listening can stimulate students' imagination and lead to greater musical achievements and breakthroughs^19,26^. In the educational development context, music listening emerged as a key antecedent of musical development^6,15^, but there is a scarcity of research on the influence of listening music willingness on musical development and achievement. Therefore, further study is needed to ascertain whether listening music willingness significantly affects the musical achievement and development of university music students.

Second, in the light of self-determination theory, music engagement is associated with autonomous form of motivation^6,27,28^, which can be intrinsic or extrinsic^29^. Extrinsic motivation for engagement may significantly affect an individual's sense of autonomy, competence, and relatedness, thereby bringing forth any potential advantages for their overall wellbeing^21^. Autonomous motivation pertains to performing a task or behavior with a sense of enthusiasm and choice^28^. Therefore, musical engagement may motivate university students for their musical achievement and development, as musical engagement involves active participation in various musical activities such as playing an instrument, singing, or composing. This exposure enhances their musical understanding and appreciation. A deeper understanding of music contributes to students' ability to interpret and perform music with greater proficiency and sensitivity^8,26^. Musical engagement often involves group activities, such as ensemble performances, choir rehearsals, or music classes^25^. These social interactions allow students to collaborate with others, learn from their peers, and develop important interpersonal skills. Musical engagement has been linked to various aspects of holistic development, including cognitive, emotional, social, and physical domains^17,30^. Active engagement with music stimulates brain development, promotes emotional wellbeing, nurtures social connections, and supports physical coordination (e.g., playing an instrument). These holistic benefits may contribute to overall student development and, consequently, their musical achievement^9^. Given the significance of music engagement, researchers have shown a growing recognition of the vital links between music engagement and reducing stress, anxiety, and depression and improving mental wellbeing during COVID^31,32^, improve life satisfaction of youngsters during pandemic^33^. However, given the novelty of music engagement, little consideration is given to the music engagement as an independent mechanism in the relationships between music engagement and musical development and achievement, which raised a number of unanswered questions, specifically regarding musical education and effect mitigation concerns. Therefore, investigating how musical engagement act as an essential precursor for students' musical achievement and development is thus necessary.

Third, generally music pleasure is based upon its aestheticism; and the aesthetic experience of music is the beauty of emotions evoked while listening to the music. Brattico et al.^22^ defined music aesthetic experience as "one in which the individual immerses herself in the music, dedicating her attention to perceptual, cognitive, and affective interpretation based on the formal properties of the perceptual experience" (p. 2). Music aesthetic experience involves a deep emotional and aesthetic connection with music^22^, and facilitating these connections to enable individuals to connect with music emotionally^12^, offering activities to foster skill development, and promoting the positive outcomes of practice are potential strategies for enhancing practice conditions^25^. Meanwhile, the act of listening to music is a prevalent behavior that is commonly practiced by individuals across various age groups, ranging from early childhood to advanced stages of adulthood and old age^30^. Therefore, music aesthetic experience and listening behavior contribute to the development of a rich musical vocabulary and interpretation skills^26^ Nonetheless, its dire need to investigate whether these factors play a prominent role in students' development and achievement. It might explore how aesthetic experiences, such as emotional connections with music or appreciation of its artistic qualities, and listening behavior, such as active and mindful engagement, contribute to students' satisfaction of their psychological needs and subsequently influence their musical development and achievement. However, despite the immense potential and significance of music aesthetic experience and music listening behavior, these crucial factors have received limited attention as mediators in the relationships between various variables and the ultimate outcome of musical development and achievement. The novelty of these concepts, coupled with their transformative influence. This knowledge gap raises numerous unanswered questions, particularly in the realm of musical education and the effective mitigation of challenges.

### Study objective and associated research questions

By utilizing a quantitative research approach, the study aims to establish an integrated model amongst listening music willingness (LMW) and Music engagement (ME) along with the mediating effects of Music listening behavior (MLB) and Musical aesthetic experience (MAE) to assess the musical development and achievement (MDA) of university music students. Therefore, based on the gaps identified above, the present study investigates the following research questions.

RQ1.: Whether listening music willingness significantly affects musical achievement and development of university music students?
RQ2.: How does musical engagement act as an essential precursor for students’ musical achievement?
RQ3.: Are music aesthetic experience and music listening behavior the most important factors in the development and achievement of university music students?

This study yields crucial contributions in multiple aspects. Firstly, it provides essential guidelines related to musical education for the upper management in universities, particularly in China. Such implications enable institutions to nurture their students' musical achievements and progress effectively. Secondly, the study unveils a novel perspective by examining the mediating role of aesthetic experience in the musical development and achievement of university music students. It sheds light on how this experience influences the overall growth of students, presenting a groundbreaking insight. Thirdly, the study highlights the importance of consistently stimulating music-listening behavior for university music students. It emphasizes whether the willingness to listen to music is insufficient to attain desired achievements. Such finding offers a new direction for students' musical development and accomplishment, underscoring the significance of intentional music-listening behavior. Moreover, the study distinguishes itself by utilizing self-determination theory as a framework, setting it apart from other theories used to investigate music related studies.

The remaining sections of the study are structured as follows: “Materials and methods” section delineates the research technique utilized to scrutinize the proposed model. “Data analysis and results” section encompasses the analysis of data and presentation of results. In “Discussion” section, the results are discussed. “Implications” section delves into the theoretical and practical implications. “Limitations and future research” section addresses future research prospects and concludes the study.

**Figure 1:**
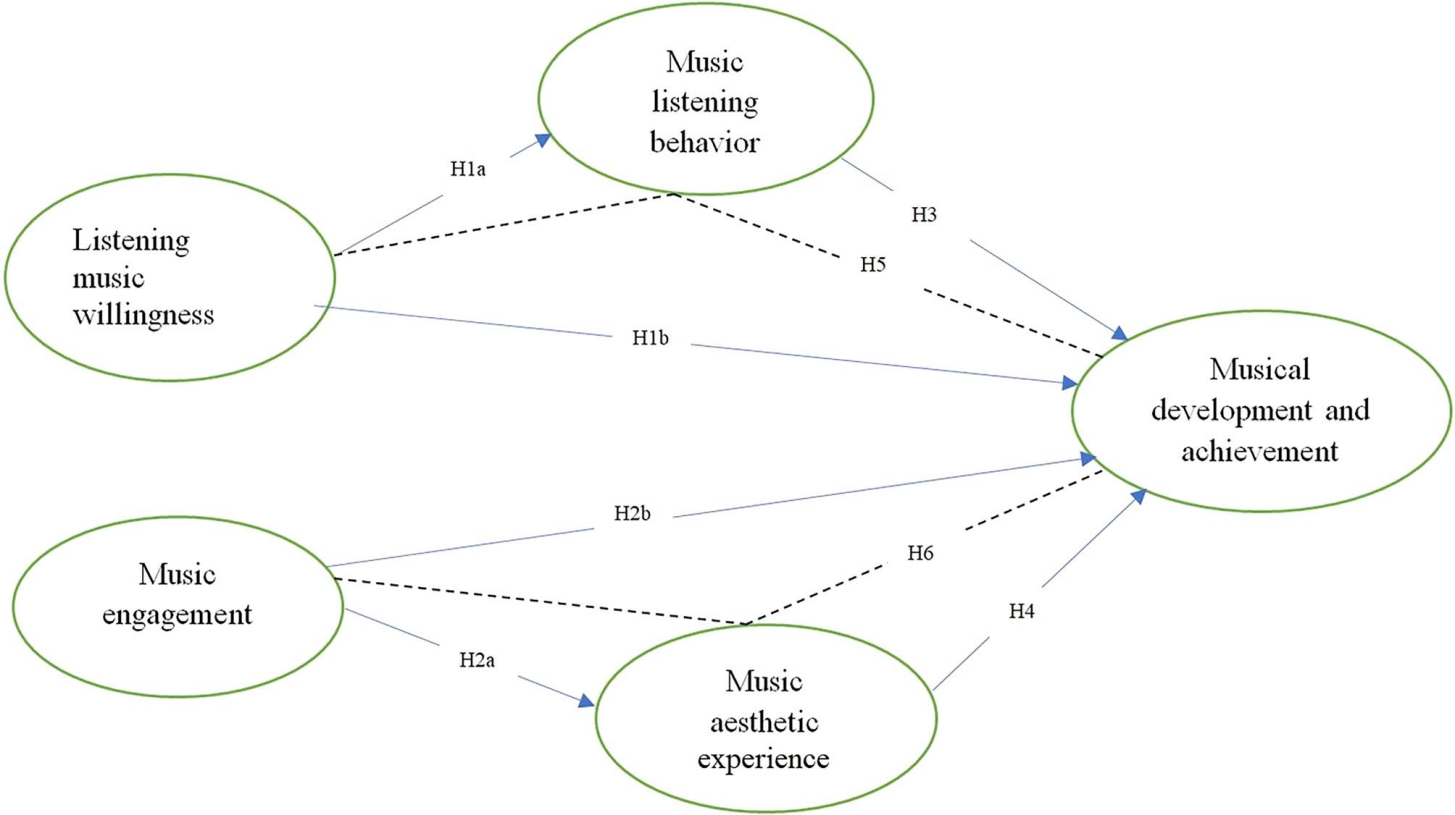
Theoretical research model.

By drawing upon self-determination theory, the proposed research model (as shown in Fig. 1) is used to explore the impact of music listening willingness and musical engagement (motivational factors) on musical development and achievement through the mediating role of musical aesthetic experience (experiential factors) and music listening behavior among university music students. To address the aforementioned research objective, the following hypotheses need to be examined.

#### Hypothesis 1a

Listening music willingness has a positive, direct and significant effect on music listening behavior.

#### Hypothesis 1b

 Listening music willingness has a positive, direct and significant effect on musical development and achievement.

#### Hypothesis 2a

 Music engagement has a positive, direct and significant effect on music aesthetic experience.

#### Hypothesis 2b

 Music engagement has a positive, direct and significant effect on musical development and achievement.

#### Hypothesis 3

Music listening behavior has a positive, direct and significant effect on musical development and achievement.

#### Hypothesis 4

Music aesthetic experience has a positive, direct and significant effect on musical development and achievement.

#### Hypothesis 5

Music listening behavior positively mediates the relationship of between listening music willingness and musical development and achievement.

#### Hypothesis 6

Music aesthetic experience positively mediates the relationship of between music engagement and musical development and achievement.

## Materials and methods

### Participants and research sample

Depending upon quantitative research methodology, convenience sampling technique has been utilized to collect data from university students having music majors. According to Malhotra^34^, a prominent illustration of convenience sampling is the use of students as study participants. Consequently, the participants of this study comprised of music students enrolled in five distinguished universities in China. An online survey platform “Questionnaire Star” (accessible at http://www.sojump.com) was utilized to gather the necessary data. Furthermore, by using the online survey the authors gathered data from university music students studying in a set of Chinese music institutions i.e. Guangxi Art University (having 700 music students), Nanjing University of Arts (comprising 600 students associated with music major), Shanghai Conservatory of Music (946 music major students), Wuhan Conservatory of Music (having 452 students with music discipline) and Xinghai Conservatory of Music (having 384 number of music students). University students have been frequently chosen as research participants in various studies related to social psychology and behavior^35–38^, especially when conducting online surveys^39^. Researchers posits that university students are a highly significant subset that is likely to be representative of a given population^40^. Consequently, the present study has chosen university music students as the study sample. The rationale behind this decision is to investigate their *musical behavior and engagement*, as it may serve as a driving force for their *musical progress and achievement*.

Initially the authors distributed the questionnaire link by sharing through different WeChat groups of 5 university music students. Furthermore, it was requested to the group members to share the questionnaire on their WeChat Moments, accordingly to get sufficient responses. This comprehensive distribution approach resulted in the collection of overall 357 responses, voluntarily. The distribution of the questionnaire was carried out with the aim of achieving the study objective.

Primarily, the authors received a total of 357 responses between April 1st and June 1st, 2023, through the online survey. The number of minimum valid responses were determined by using the formula n = 5 × m, where "m" represents the number of items, as established by Comrey et al.^41^ and Worthington and Whittaker^42^. Based upon aforementioned formula, the minimum valid sample size should be 125, as there are 25 study items in this research. However, irrespective of the approach employed, failure to recognize the fundamental principles of sampling theory yields meaningless outcomes^43^. Of the total responses received, 299 were deemed valid, indicating that the sample size of *n* = 299 is adequate for conducting this research endeavor. Besides, to make the data valid and ready to analyze, the authors excluded incomplete questionnaires, deleted repeated responses and removed questionnaires having missing values.

The demographic information of the participants is presented in Table 2. The study comprised a substantial proportion of male participants, with a total of 180 individuals representing 60.20% of the sample.

**Table 2:** Demographic of respondent (*N* = 299).

Characteristics		Frequency proportion	Proportion%
Gender	Female	119	37.7
	Male	180	60.20
Age	15 to 20 years	160	53.5
	21 to 25 years	89	29.30
	26 to 30 years	40	13.37
	31 to 35 years	10	3.34
	35 and above	0	0
Education	Short course	33	11.03
	Bachelors	174	58.19
	Masters	79	26.42
	PhD	13	4.34
University/institution	Guangxi Art University	74	24.66
	Nanjing University of Arts	61	20.33
	Shanghai Conservatory of Music	90	30
	Wuhan Conservatory of Music	40	13.33
	Xinghai Conservatory of Music	35	11.66
Source of music	Youku	25	8.33
	QQ	98	32.66
	YouTube	39	13.00
	e-Clouds	51	17
	Other	87	29
Daily, time consumed to engage with music activities	Less than 1 h	64	21.33
	1–2 h	39	13
	2-3 h	67	22.33
	3–4 h	26	7.66
	4–5 h	46	15.33
	More than 5 h	60	20

Majority of the respondents, comprising 160 individuals or 53.5%, fall within the age range of 15 to 20 years. In terms of the educational background of the participants, it was found that 174 individuals, comprising 58.19% of the respondents, were pursuing their bachelor's degree. The remaining survey participants were engaged in various educational pursuits such as master's degrees, short musical courses, and PhD programs. Regarding the source of data collection, the majority of participants were affiliated with Shanghai Conservatory Music institution, comprising 90 individuals or 30% of the sample. The remaining participants were affiliated with other universities and institutions, including Guangxi Art University (*n* = 74, 24.66%), Nanjing University of Arts (*n* = 61, 20.33%), Wuhan Conservatory of Music (*n* = 40, 13.33%), and Xinghai Conservatory of Music (*n* = 35, 11.66%), respectively. Regarding the source of music among the participants, it was found that 98 individuals, accounting for 32.66% of the sample, relied on the QQ music application. This was followed by YouTube, e-Cloud, and other sources, in descending order of frequency. Out of all the participants, the maximum number of survey participants, which is 67, reported spending 2 to 3 h daily engaging in music-related activities, representing 22.33% of the total sample.

Furthermore, Hair et al.^44^ suggest that prior to the use of SEM, it is necessary to figure out the sample size criterion through power analysis. Therefore, this study employs a priori sample size calculator for structural equation modeling (SEM), as developed by Soper^45^. The utilization of this calculator necessitates a set of input data, including the anticipated effect size, levels of statistical power, the number of observed variables (items) and latent variables (study constructs) in the model, and the preferred probability for detecting the minimum sample size for SEM technique, as outlined in Westland^46^. The study's parameters were defined by inputting specific values, including a desired statistical power level of 90 percent, five unobserved constructs, 25 observed variables, a probability level of 0.05, and an anticipated effect size of 0.3. The recommended minimum sample size is 188 and the minimum sample size for model structure is 100. Therefore, the aforementioned criterion has been satisfied, given that the current study's authentic and acceptable sample size is 299.

### Ethical approval

All materials and methods are performed in accordance with the instructions and regulations and this research has been approved by a committee at Guizhou Minzu University, China. This research has been approved by the Institutional Review Board at Guizhou Minzu University, China. All procedures performed in studies involving human participants were in accordance with the ethical standards of the institutional research committee and with the 1964 Helsinki declaration and its later amendments or comparable ethical standards.

### Participant consent statement

To address the fundamental research ethical principles informed consent was obtained from all participants included in the study, which they read before proceeding the survey form. The document included ethical rules and information regarding data processing, outlining the study's objective, the estimated duration of the survey, caring for personal information, and clarifying voluntary participation, as well as the right to discontinue participation in the survey at any point. In order to safeguard the integrity of study participants, the study refrained from utilizing any personal or confidential information, thereby ensuring anonymous participation.

### Instruments

The survey instruments and items have been adapted from highly cited research articles that have tested related survey instruments. Some questionnaire items were slightly altered.

#### Listening music willingness

Listening music willingness is the first independent variable and the associated items were taken from^47^. This variable comprises 5 items, measuring the impact of *listening music willingness on music listening behavior* and *musical development and achievement* among university music students.

#### Music engagement

This instrument is the study’s second independent variable contains 5 reflective items which entail the amount of time spent by music students to listening music. *Music engagement* was evaluated by drawing on the items from Rosenberg et al.^48^ and Schäfer and Eerola^11^.

#### Music aesthetic experience (MAE)

MAE is the first mediating variable that examines the mediating relationship between *listening music willingness* and *musical development and achievement* in this study. *Music aesthetic experience* has been measured by the items taken from Reybrouck et al.^13^. Five reflective items can reflect the outcomes due to which an individual may aesthetically motivate towards *musical development and achievement*.

#### Music listening behavior (MLB)

*Music listening behavior* is the second mediating variable that entails the indirect relationship between *music engagement* and students’ *musical development and achievement*. This construct contains five questionnaire items taken from Behbehani and Steffens^7^.

#### Musical development and achievement (MDA)

*MDA* is the final dependent variable of this study. The items for *musical development and achievement* were taken from Oliveira et al.^9^ Five items measure the *achievement and development* among university music students.

All of the items were assessed on a *five-point Likert scale* that ranged from *1* = *strongly disagree, 2* = *disagree, 3* = *neither agree nor disagree, 4* = *agree, 5* = *strongly agree*. The reason to use *five-point Likert scale* is the most accurate type among other scales to gather and analyze Likert scale data^49^. It gives a better image of the respondent's true opinion. To confirm a general reliability check, the questionnaire items and instruments were cross-checked by five PhD students related to music behavior. Subsequently, two professors reviewed the questionnaire for further corrections and authentication.

### Procedures

In this study the research model has been examined by using structural equation modeling (SEM) analysis technique. There are many justifications for using SEM. For instance, running a regression with mediation is preferable^50,51^. To ensure the questionnaire terms and wording were clearly understood by the respondents, first, we administered a pilot study among 50 music students with a ratio of 6 male and 4 female music students enrolled in each selected university. Among them, 23 students were doing bachelor, 10 students were engaged with their master’s degree and rest of them were doing short certifications and PhD. In term of respondents’ age for the pilot study, majority of the pilot study respondents’ age is 20 years or less i.e. 21 respondents; followed by remaining age groups such as 21 to 25 years i.e. 14 students. Students having age from 26 to 30 years were 8, 7 students have their age from 31 to 35 years, respectively. Second, By following Hinkin^52^ suggestion to ensure the reliability and validity of the chosen items, the authors check the statistical reliability of the pilot study in the form of internal consistency of the constructs and the items, having a range of 0.717–0.868 for the internal consistency of the variables, which satisfies Hair et al.^53^’s minimal value requirement of 0.7. Keeping in view the responses to the pilot, we slightly modified few items’ wordings. Subsequently, two professors reviewed the questionnaire for further corrections and authentication. The pilot and main survey of the study were conducted as early as possible, and a minimal time gap was maintained so that it did not create any impact on the study.

The data obtained using a survey method have been effectively and efficiently evaluated by using SEM method^54,55^. SEM has the capacity to evaluate correlations between latent and observable variables at the same time^56^. Additionally, SEM computes measurement error and offers a precise estimation of the mediation effect^57^. Furthermore, it is suitable for both complex and basic research architectures and does not necessitate computing data normality^53^.

Prior to conducting an effective SEM data analysis procedure, the authors performed a non-response bias test on the research sample with regards to gender. Second, a multicollinearity test was conducted to assess potential correlation and covariance concerns among the variables, given that the data was gathered through an online survey. Thirdly, the authors have assessed the overall fitness of the model through the utilization of various statistical techniques. The statistical measures of chi-square, comparative fit index (CFI), root mean square error of approximation (RMSEA), and Tucker-Lewis index (TLI) are commonly utilized in academic research. Fourth, a confirmatory factor analysis (CFA) was conducted to authenticate the measurement model. Subsequently, a structural equation model (SEM) was executed to examine the interrelationships among the constructs. In addition, mediation analysis among the constructs was conducted by following the Baron and Kenny^58^ and Rucker et al.^59^ recommendations. According to Baron and Kenny^58^ criteria, the following conditions must be fulfilled to identify a mediation effect: (1) If the relationship between the independent variable and dependent variable is statistically significant and, at the same time, the mediating relationship is also significant, it will be considered partial mediation. (2) If the relationship between the independent variable and dependent variable is statistically insignificant and the mediating relationship is statistically significant, it will be considered full mediation. Besides, Rucker et al.^59^ noted that “mediation analyses focus on examining the magnitude of indirect effects,” and such effects are best accessed via SEM analyses. Thus, this paper employed SEM and then used bootstrapping tool to analysis the valid data. All the statistical tests mentioned in the study were conducted with the help of SPSS 24 and AMOS 24 software.

## Data analysis and results

### Non response bias

The authors conducted an analysis of potential nonresponse bias, utilizing the approach established by Armstrong and Overton^60^. The authors of this study partitioned the research sample into three equal parts, namely the first one-third, the second one-third, and the third one-third. By following the patterns of previous studies, this study conducted a non-response bias test by examining the gender distribution (male and female) of the first one-third and last one-third samples^61,62^. Based on the findings, it appears that there were no statistically significant gender disparities between the two groups, as indicated by the p-values ranging from 0.16 to 0.58. Thus, the nonresponse bias of the present study has been eliminated.

### Multicollinearity test

Prior to conducting a structural equation modeling (SEM) analysis, it is imperative to assess the presence of multicollinearity constraints. According to Abraham et al.^63^ the presence of a strong correlation between two or more exogenous variables leads to the phenomenon of multicollinearity. Variance inflation factor (VIF) and tolerance are two widely used measures for assessing multicollinearity^53^. The Variance Inflation Factor (VIF) represents the initial metric for assessing the presence of multicollinearity. According to Bhukya and Singh^64^ if the VIF value is below 3.0, it is assumed that there is no problem of multicollinearity. The VIF values of the present investigation ranged from 1.788 to 2.232, with an acceptable value of 3.0. The threshold tolerance values reported by Kutner et al.^65^ ranged from 0.1 to 1.0. The study's tolerance values fall within the acceptable range, with a range of 0.455 to 0.560. Consequently, it has been ascertained that this study does not exhibit any multicollinearity concerns.

### Measurement model

The evaluation of the measurement model involved assessing the overall model fit, construct reliability, and validity. To account for sample size sensitivity, the ratio of the chi-square statistic (χ^2^) to the degrees of freedom (*df*) was used. The resulting value of χ^2^/*df* = 1.54 (χ^2^ = 268.85, *df* = 174) was below the acceptable threshold of 3, as suggested by Teo and Zhou^56^. The comparative fit index (CFI), recommended by Koufteros^66^ should be at least 0.9 to indicate adequate model fit, and in this case, the CFI was 0.968. The root mean square error of approximation (RMSEA) should be below 0.08, according to Teo and Zhou^56^ while MacCallum et al.^67^ consider RMSEA values below 0.05 as "good". For the measurement model in this study, the RMSEA was calculated as 0.043. Considering all the fit indices, it can be concluded that the final model fits reasonably well with the sample data.

**Figure 2:**
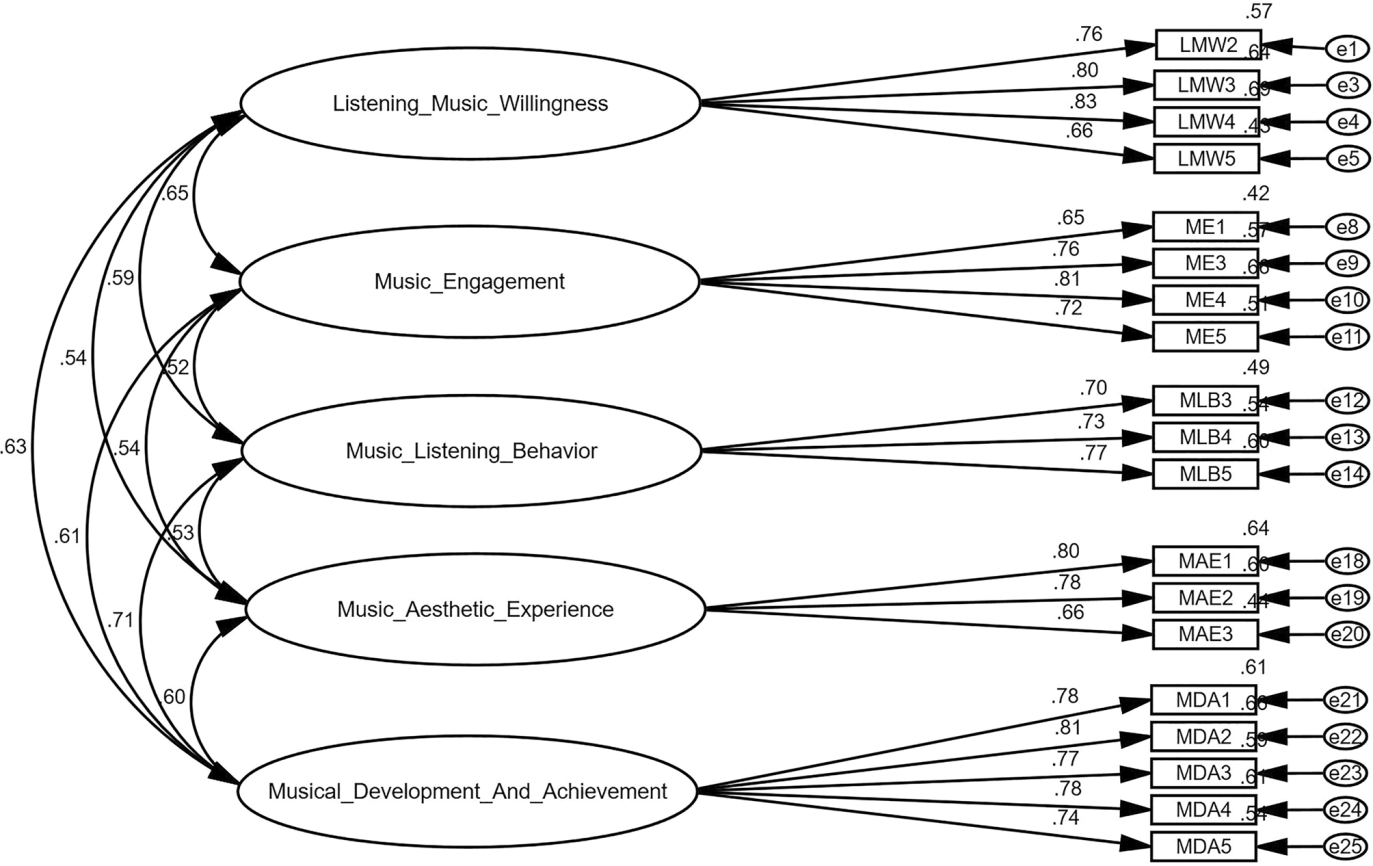
Measurement model/CFA.

The measuring model includes convergent validity, discriminant validity, internal consistency reliability, and item-by-item reliability. According to Table 3, the lowest loading is 0.65 and the maximum is 0.83, as shown in Fig. 2, both of which are larger than the 0.50—a threshold value for each factor loading^53^. Cronbach's alpha and composite reliability (CR), which measure internal consistency and reliability, were calculated^57^. Researchers identified that CR works better while conducting SEM. In this research, the above-mentioned conditions of reliability tests were fulfilled. The Cronbach’s alpha ranging from 0.800 to 0.879 and CRs from 0.806 to 0.879. Table 3 reveals that the measurement model was internally consistent and reliable because the CR value and Cronbach's alpha for all latent variables (LVs) are greater than 0.70. Convergent validity and discriminant validity were both functions of the variable validity of the reflective measurement model.

**Table 3:** Confirmatory factor analysis.

Constructs and items	Factor loading	SMC	CR	Cronbach's α	AVE
Listening music willingness (LMW)			0.848	0.843	0.583
LMW 2	0.76	0.57			
LMW 3	0.80	0.64			
LMW 4	0.83	0.69			
LMW 5	0.66	0.44			
Music engagement (ME)			0.824	0.818	0.541
ME 1	0.65	0.42			
ME 3	0.76	0.57			
ME 4	0.81	0.66			
ME 5	0.72	0.51			
Music listening behavior (MLB)			0.780	0.778	0.542
MLB 3	0.70	0.49			
MLB 4	0.73	0.54			
MLB 5	0.77	0.60			
Music aesthetic experience (MAE)			0.790	0.786	0.558
MAE 1	0.80	0.64			
MAE 2	0.78	0.66			
MAE 3	0.66	0.44			
Music development and achievement (MDA)			0.883	0.882	0.602
MDA 1	0.78	0.61			
MDA 2	0.81	0.66			
MDA 3	0.77	0.60			
MDA 4	0.78	0.61			
MDA 5	0.74	0.54			

*CR* composite reliability, *SMC* square multiple correlation, *AVE* average variance extracted.

The discriminant validity and construct validity in the model were also evaluated. The benchmark of construct validity assessments are: (1) all the factor loadings must be greater than or equal to 0.60^68,69^. (2) The value of CR of each construct must be at least 0.70 or above^57^. (3) The average variance extracted (AVE) value should be at least 0.50^70^. After removing items *(LMW1, ME2, MLB1, MLB2, MAE4 and MAE5)* created issues while calculated AVE, all other remaining constructs and items contented the required conditions for a good convergent validity. The CR values of all items ranging from 0.78 to 0.883, AVE values from 0.541 to 0.602 and all factor loading values ranging from 0.65 to 0.83 showing good convergent validity. For a decent square multiple correlation (SMC), the value of all items must be greater than or equal to 0.36, because SMC articulates, how well an item measures a construct^69,71^. In term of SMC this study has values ranging from 0.42 to 0.69 (see Table 3).

### Discriminant validity analysis

Every variable is statistically different from other variable, which is referred to as having discriminant validity^72^. Prior to a few decades ago, Fornell and Larcker^70^ standard criteria were used to quantify discriminant validity, which is now considered as outdated. Researchers developed a new method to calculate discriminant validity called as heterotrait-monotrait (HTMT)^69^. Furthermore, according to Henseler et al.^69^, HTMT is regarded as a suitable technique when loading has a slight difference. The HTMT value is 0.90 for LVs with the same concept and 0.85 for LVs with a different concept^73^. The value of all LVs is less than 0.85, as shown in Table 4, satisfying the discriminant validity condition.

**Table 4:** Discriminant validity.

	LMW	ME	MLB	MAE	MDA
Listening music willingness (LMW)					
Music engagement (ME)	0.676				
Music listening behavior (MLB)	0.610	0.547			
Music aesthetic experience (MAE)	0.568	0.570	0.547		
Musical development and achievement (MDA)	0.642	0.626	0.705	0.613	

Thresholds are 0.850 for strict and 0.900 for liberal discriminant validity.

### Structural model/hypotheses testing

**Figure 3:**
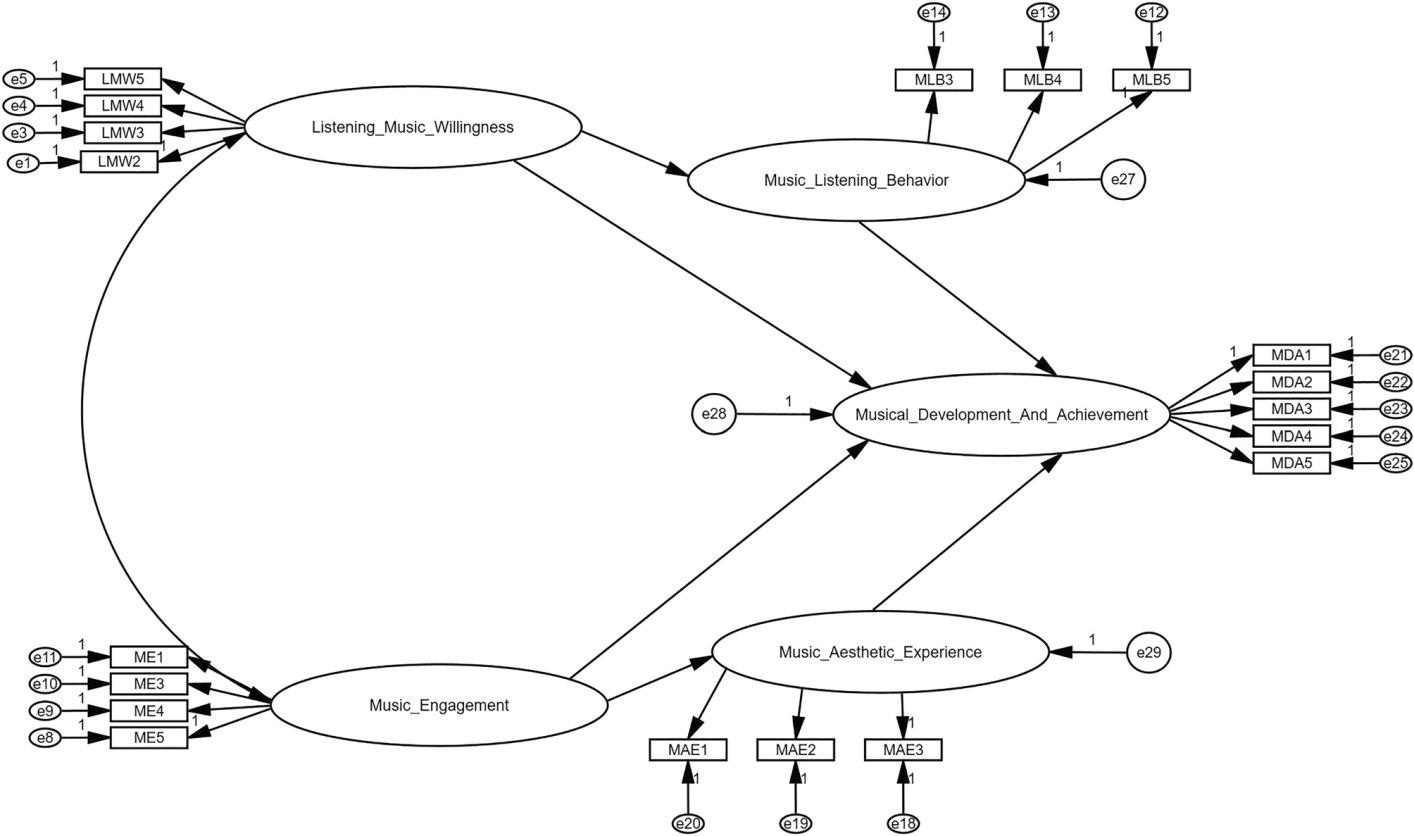
Research model to conduct SEM along with all qualified constructs and items.

**Figure 4:**
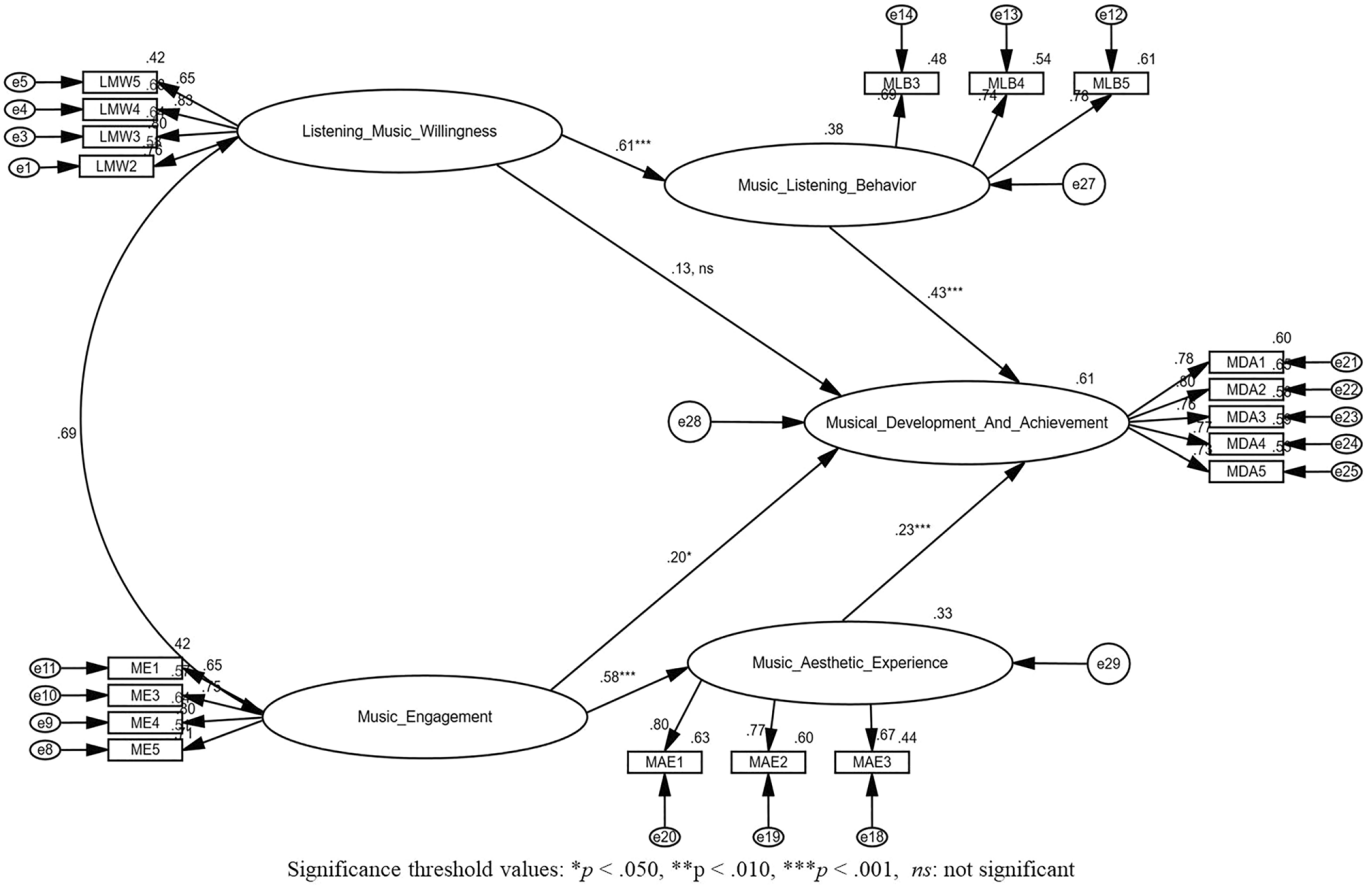
Structural model results.

To perform hypotheses testing the author has carried out structural equation modeling with the help of AMOS 24. The path diagram is shown below in Fig. 3. The results of structural equation model (SEM) test are presented in Fig. 4. According to the research model, all direct and indirect hypotheses are significant, except the direct relationship between music engagement and music development and achievement. Figure 4 presents detail information as follow.

#### Direct relationships

*Listening music willingness* has a significant and positive effect on *music listening behavior* (β = 0.61***, p < 0.001), hence, supporting H1a. On the other hand, according to hypothesis 1b the direct relationship between *listening music willingness* and *musical development and achievement* is insignificant (β = 0.13, ns, p ≰ 0.05), hence H1b is unsupportive. Furthermore, H2a which denotes *music engagement* has significant, positive and direct effect on *music aesthetic experience* (β = 0.58***, p < 0.001), Hence the author has declared H2a as supportive and significant. H2b which corresponds *music engagement* has a significant effect on *musical development and achievement* (β = 0.20*, p ≤ 0.05). According to H3, *music listening behavior* is positively, significantly and directly influence *musical development and achievement* (β = 0.43***, p < 0.001). According to H4, the direct relationship between *music aesthetic experience* and *musical development and achievement* is significant and positive (β = 0.23***, p < 0.001). As Table 5 presents hypotheses testing results.

**Table 5:** Direct hypotheses testing.

Hypotheses	Relationship	C.R (t-value)	*P*	Standardized structural coefficients (β)	Interpretation
H1a	LMW → MLB	7.730	0.001***	0.61***	Significant
H1b	LMW → MDA	1.510	0.131	0.13, ns	Insignificant
H2a	ME → MAE	7.342	0.001***	0.58***	Significant
H2b	ME → MDA	2.340	0.019*	0.20*	Significant
H3	MLB → MDA	5.231	0.001***	0.43***	Significant
H4	MAE → MDA	3.371	0.001***	0.23***	Significant

*LMW* music listening willingness, *ME* music experience, *MLB* music listening behavior, *MAE* music aesthetic experience, *MDA* music development and achievement, *ns* not significant.

Significance threshold values: *p < 0.050, **p < 0.010, ***p < 0.001.

#### Mediating/indirect relationships

**Figure 5:**
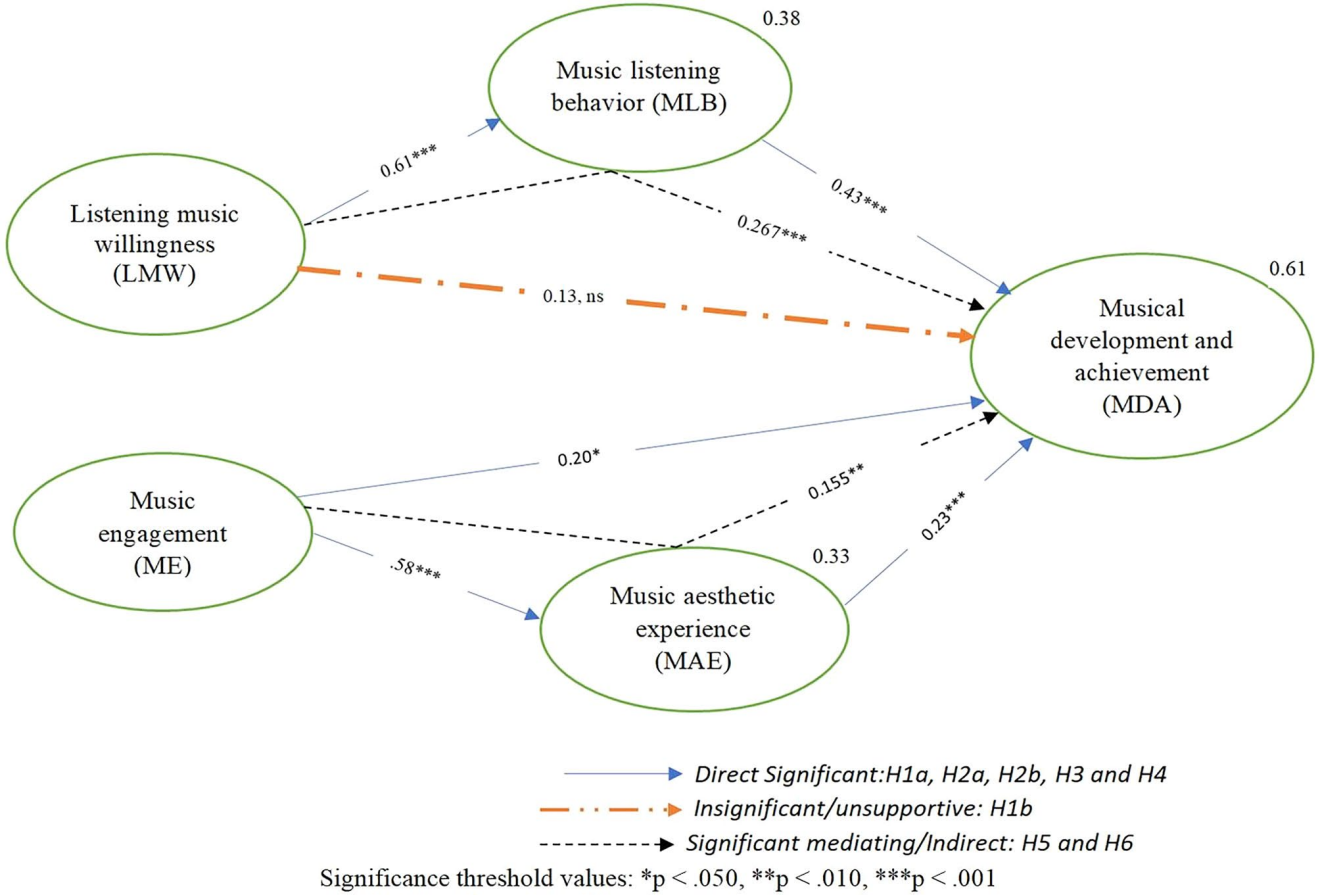
Structural model with direct and mediating/indirect hypotheses.

The authors of this study tested indirect/mediation effect using the guidelines of Baron and Kenny^58^ and Rucker et al.^59^ recommendations. To conduct an indirect/mediation analysis the author has used the bootstrapping tool by fixing 95% confidence interval (CI) and 5000 number of bootstrap samples. The results are shown in Table 6 and Fig. 5, reported that the required mediation conditions of Rucker et al.^59^ and Baron and Kenny^58^ were fulfilled and satisfied.

**Table 6:** Indirect/Mediating hypotheses testing.

Hypothesis	Relationship	Total effect	Direct effect	Indirect effect	Confidence intervals		P value	Interpretation
					Lower bounds (BC)	Upper bounds (BC)		
H5	LMW → MLB → MDA	0.41	0.13	0.267	0.140	0.451	0.001***	Full mediation
H6	ME → MAE → MDA	0.39	0.58	0.155	0.041	0.341	0.009**	Partial mediation

*LMW* listening music willingness, *ME* music engagement, *MLB* music listening behavior, *MAE* music aesthetic experience, *MDA* music development and achievement.

Significance threshold values: *p < 0.050, **p < 0.010, ***p < 0.001.

*Listening music willingness* (independent variable) has correlated and mediated the relationship with *musical development and achievement* (dependent variable), in the presence of *music listening behavior* (mediator) (β = 0.267***, p < 0.001). In addition, there is no zero between the values of lower bound (BC) (0.140) and upper bound (BC) (0.451). Hence H5 presents full mediation relationship. Because, the direct relationship between *listening music willingness* and *musical development and achievement* is insignificant and unsupportive i.e. (β = 0.13, ns, p ≰ 0.001). Therefore, the author has anticipated that H5 presents full mediation relationship between *music listening willingness* and *musical development and achievement* through *music listening behavior*. Furthermore, the author found that the relationship between *music engagement* and *musical development and achievement* has also been positively mediated i.e. (β = 0.267***, p < 0.001) due to the existence *music aesthetic experience* as mediator between the dependent and independent variables. Besides, there is no zero between the values of lower bound (BC) (0.041) and upper bound (BC) (0.341). Hence the author found that H6 is significant and brings forth partial mediation between the independent variable i.e. *music engagement* and dependent variable i.e. *musical development and achievement* via mediating variable – *music aesthetic experience*, because, the direct relationship between *music engagement* and *musical development and achievement* is also significant and supportive i.e. (β = 0.58***, p < 0.001).

Hence it is proved that both of the indirect/mediating hypotheses are positive and significant, as shown in Table 6.

Finally, the value of R^2^ on music listening behavior is 0.38 and the value of R^2^ on music aesthetic experience by the music engagement is 0.33 (see Fig. 5). Furthermore, the total combine variance exerted by all variables including depended and mediating variable on the final depend variable—musical development and achievement is 0.61, 61 percent. For more details, see Fig. 5.

## Discussion

Current study investigates three pivotal research questions: whether listening music willingness significantly effects musical achievement and development of university music students? How does musical engagement act as an essential precursor for students’ musical achievement? Are music aesthetic experience and music listening behavior the most important factors in the development and achievement of university music students, and if so, how? These research questions align with self-determination theory by considering the role of autonomy, competence, and relatedness. This research focuses on the impact of music listening willingness and music engagement, which foster musical development and achievement through the mediating role of musical aesthetic experience and listening music behavior among university music students.

First, according to H1a, the direct relationship between listening music willingness (LMW) and music listening behavior (MLB) is positive and significant. The empirical findings clarify that positive intention, thinking, or attitude towards music significantly contributes to stimulate university students’ music motivation in the form of actual music listening behavior from different sources. The outcomes are inline-with and verify the findings of Kamalzadeh et al.^74^, in which musical listening willingness significantly determines music listening and managing behaviors, which could also be influenced by other demographic factors. On the other hand, this study reveals that there is neither a significant, positive, nor direct relationship between listening music willingness and students’ musical developments and achievements (MDA), thus, unsupportive H1b. The study result contradict with the findings of Leung and Cheung^20^, they examined Chinese adults’ engagement with music and social-emotional achievement in the form of well-being. They found that there is a positive, significant, and direct association between music listening readiness/willingness and (social-emotional) development and achievement. Whereas this study findings claim that there is no any direct and no any significant relationship between listening music willingness and students’ achievement and development, because, there might be few other factors which act as mediators or moderator between listening music willingness/readiness and students’ achievements and developments. A potential explanation is that students who demonstrate a higher willingness to listen to music are more inclined to adopt consistent listening behaviors.

However, it is important to note that this willingness alone may not fully contribute to their musical development and achievement. While listening to music is undoubtedly enjoyable and enriching, it may not directly translate into tangible progress or notable accomplishments in their musical pursuits. Besides, individuals who have a higher willingness to listen to music are more likely to engage in music listening behavior. This finding aligns with the concept of intrinsic motivation in SDT, as individuals with a strong intrinsic motivation for music are more likely to voluntarily engage in music listening as a satisfying and enjoyable activity. While intrinsic motivation for listening to music can drive individuals to engage in the behavior, the development and achievement in music require additional factors beyond mere willingness.

Second, music engagement (ME) has a significant influence on music aesthetic experiences (MAE) and supports H2a. This study illuminates that engagement in musical activities (i.e., listening music, playing instruments, creating music rhythms, etc.) significantly contributes to the advancement of pleasant experiences for university music students. These results are similar to Hallam^75^ finding, that the positive effects of engagement with music develop an enjoyable and rewarding experience, particularly among young individuals. Furthermore, this study reveals that there is significant, direct, and positive relationship between musical engagement and students’ musical developments and achievements (MDA) thus, supporting H2b. The outcomes are in line with and verify the findings of Jian^15^ while examining the relationship of engagement and academic achievement within music students; according to his findings, student sustainable engagement has a significant and positive relationship with student academic achievement^15^. As this study's findings also claim, there is a significant relationship between music engagement and students' achievement and development. The compelling explanation is that music engagement is undeniably linked to both the musical aesthetic experience and the overall development and achievement of students.

Numerous studies have consistently demonstrated a significant correlation between music engagement and the profound aesthetic experiences students encounter. Engaging actively with music allows students to immerse themselves in the emotional depths, connect with the artistic expressions, and truly appreciate the captivating beauty of music. Moreover, this engagement serves as a powerful catalyst for their musical development and achievement. By actively participating in various musical activities, such as practicing, performing, and composing, students not only hone their skills and expertise but also foster consistent engagement-oriented as well as a sense of competence, by the means of self-determination theory. This invaluable experience empowers them to reach new heights, overcome challenges, and ultimately achieve remarkable success in their musical pursuits.

Third, this research suggests that music listening behavior is considered a crucial role in attaining students’ musical developments and achievements, hence, supporting H3. The results verify Faran et al.^16^’s outcomes, who found that music listening activities positively and significantly lead to students’ achievements in Pakistani universities. It is because, among young people, music is considered as source of refreshment or alternate way for mind peace, and mental peace brings forth success and achievements in every perspective. Moreover, as viewed through the lens of self-determination theory, exerts a profound influence on the musical development and achievement of university music students. By satisfying their need for autonomy, competence, and relatedness, active music listening becomes a catalyst for their intrinsic motivation, skill development, and social integration within the music community. Embracing and nurturing this behavior can unlock the full potential of students, propelling them towards exceptional musical growth and achievement.

Fourth, music aesthetic experience (MAE) has a significant positive influence on the musical development and achievement of university students and is supported by H4. This study reveals that students’ prior joyful music experiences significantly assist in attaining their achievements and developments in Chinese musical institutions. If these musical institutions and universities want to attain advancements in students' musical achievements in the long run, then there is a need to focus on students’ musical aesthetic experiences. Moreover, recently, researchers found that music aesthetic experience is a powerful determinant to stimulate musical achievement, development, and performance. Scholars found that the aesthetic experience as the main determinant of enrollment and retention in adult community choirs (a group of singers who are organized and perform together, typically during church services or other public events) that require auditions^25^. Additionally, music aesthetic experiences, viewed through the lens of self-determination theory, play a pivotal role in the musical development and achievement of university music students. By nourishing their need for autonomy, competence, and relatedness, these experiences ignite students' intrinsic motivation, enhance their musical proficiency, and foster a sense of belonging within the musical community. Embracing and nurturing music aesthetic experiences empowers students to embark on a transformative musical journey, unlocking their full potential and propelling them towards exceptional growth and accomplishment.

According to the study's 5th hypothesis, music listening behavior significantly mediates the relationship between listening music willingness and students’ musical development and achievements (MDA) thus, supporting H5. Because, the evolution, positivity, and growth in university students’ musical development and achievements are linked with their music listening behavior, as nowadays, every youngster, particularly university students, are listening music most of the time, even by indulging with other physical works or during their rest time. These results are aligned with the findings of Mohan and Thomas^76^ who examined the relationships between background music and the cultural inclination to music on adolescents’ task accomplishments, their outcomes revealed that listening background music significantly and positively mediates and enhances adolescents' listening willingness and achievement on reading comprehension activity. The probable explanation in the light of self-determination theory music listening behavior serves as a manifestation of their willingness to explore, discover, and connect with the diverse world of music. In turn, this autonomy-driven behavior fuels their intrinsic motivation and fosters a deep passion for music, setting the stage for their musical development.

Finally, music aesthetic experience significantly and fully mediates the relationship between music engagement and students’ musical development and achievements, thus supporting H6. The aforementioned findings are in line with Hallam^75^, who examined the impact of young people music engagement on their personal development has been examined. His study implies that the benefits of engagement with music for personal and social development only manifest if doing so is a pleasurable and satisfying experience. Hence the author of this study anticipates that music aesthetic experience is one of the main essential mediators that can strengthen the relationship between students’ engagement with music and their achievements and development. This interpretation is consistent with the results of Loepthien and Leipold^10^, who revealed that musicians with high degrees of musical experience strengthened and highlighted the association between music flow (A state of total engagement in music), subjective well-being, and accomplishment while engaging in musical activity.

It concludes that the relationship between musical accomplishments and musical involvement will be greatly mediated by musicians with higher levels of musical aesthetic experience. Higher levels of musical experience also boost a musician's self-confidence, which serves as a catalyst for achieving a desired outcome^26^. Overall, music engagement serves as the catalyst for students' active involvement in various musical activities such as practicing, performing, and composing. This engagement demonstrates their autonomy, as they enthusiastically invest their time and effort in the pursuit of their musical passions and interests. As students wholeheartedly immerse themselves in the musical journey, they open themselves up to captivating and emotionally resonant music aesthetic experiences. Such experience encompasses the profound emotions, connections, and appreciation that students encounter as they engage with music. These experiences awaken their intrinsic motivation and ignite a deep passion for music with melodies, harmonies, and rhythms, which ultimately encourage the students for their exceptional musical growth and achievement.

## Implications

### Theoretical implications

In previous studies, musical achievement and development of music students via music listening, music engagement, and music aesthetic experience had rarely been discussed together. This study extends^15^, who found that student sustainable engagement has a significant and positive relationship with student academic achievement. Hence, based upon self-determination theory, this study incorporates music listening willingness, music engagement, music listening behavior and musical aesthetic experiences to predict university students’ musical achievement and development. The research model offers a gorgeous insight into the double role of listening music willingness/readiness and music engagement in examining music listening behavior and music aesthetic experience which collective influence students’ musical achievement and progress. Music engagement and listening music willingness have a stronger influence on students’ achievements in light of music aesthetic experience and listening music behavior. Besides, the outcomes reveal that music listening behavior and music aesthetic experiences assist university music students to gain optimal progress and success which leads to the acquisition of rewards, good grades, creativity in musical domain and intrinsic satisfaction.

Prior researchers identified the effect of musical willingness on desirable goal achievements^74^. This study outcome reveals that there is no any significant direct relationship between music listening willingness and musical achievement; as music listening behavior has play a substantial mediating role between students willingness towards music and their musical achievements. Accordingly, the university musical students may not succeed in getting their desirable achievements until they concentrate to stimulate their music listening behavior, consistently. Musical engagement is a multidimensional phenomenon. Halliday^18^ measure musical engagement through preferences and uses, but the scant concentration is paid over complete musical engagement. Thus, the authors of this study anticipate that the multifaceted musical engagement composed of songs creation, rhythm formation, instrument playing, music storing, music sharing via diverse media etc.

This study presents another theoretical contribution by using self-determination theory instead of using theory of music or learning music theory like other researchers followed this in a similar perspective^20^. As the authors believe that the student’s achievement and development is sort of final incentives which is totally interlinked with the students’ motivations, behaviors, experiences and his intend or readiness. Therefore, the more focus on students’ motivations and intend the more achievements will be availed. Prior researchers used music listening activity to measure health issues or pain relief mechanism^77,78^, particularly for health workers during COVID pandemic^32^ and ignore its effect on the achievement and development phenomenon; they do not pay attention to any theory in building that relationship^79,80^. Hence, this study covered this gap and using self-determination theory in building the relationship among music listening willingness, music engagement, music listening behavior, music aesthetic experience and university students’ musical achievements and development. According to this theory, meaningful activities that meet people's psychological needs can promote human motivation, development, and wellness^21^. It also demonstrates how people have a natural desire to strive towards growth in order to obtain either intrinsic or extrinsic rewards.

### Practical implications

From a managerial perspective, this study results play a crucial role in attaining better achievements and developments of university music students through musical aesthetic experience, music listening behavior, music listening intent/willingness and over all music engagement. If the upper management of universities, particularly universities in China wants to attain their students’ musical achievements and progress. They need to introduce monthly based appreciation and acknowledgement policies. The management need to appreciate music students who are skillful, clever, creative, distinct in their responsibilities/punctual, exerting their efforts to develop new music ideas and knowledge, those music students who are putting their efforts to progress academically as well as partially. To do so, the policy makers need to give rewards and incentives tangibly and intangibly. Such policies will stimulate music students’ motivations and their inner satisfaction and self-confidence which holistically boosts students much engagement and their music behavior. This ultimately stimulate their achievements and progress during their stay in school as well as after graduation.

Moreover, the music related school and institutions must have processes for exchanging music knowledge with music production house/studios and partners. Acquiring and sharing knowledge about new music products and events among sister institutions would influence students’ music engagement and music behavior which may leads positivity in aesthetic experience and students’ achievements and development simultaneously. Besides, the music curriculum must be composed of less theoretical and much practical. The reason is that such policies will influence students’ satisfaction level and they will express an optimistic behavior towards practical music engagement which may results positivism in their musical achievements and success.

Furthermore, music playing has a strong relationship with training and practice. Therefore, the institutions and the stake holders need to launch music training and practice sessions consistently. Because, the students’ musical aesthetic experience will be enhanced and this strengthened experience will certainly influence their musical achievements positively at their institutions and even after completing their studies by composing and creating new music rhythms, beats, and other musical product.

The policy makers and the music school management need to launch a centralized online music library platform where the music student can join by using his/her student number and can listen latest musical tunes, rhythm, instruments, songs, beats, etc. 24/7. Because, the university students have ample of option to stay tune with music listening activity most of the time. Such a policy will certainly stimulate university students’ music listening readiness which can positively affect their music listening behavior too, and have a productive resultant effect of their achievements. Because, it has been found that there is a positive association between music listening, training and individuals’ gradual progress. Furthermore, to increase the student’s motivation towards musical success and achievements, the musical schools need to invite renown national and international music personnel by arranging interactions sessions with students. This will stimulate students’ internal motivation and compel them intentionally and unintentionally to listen music sessions where their ideals or liked ones are performing. Such a music listening stimulation policy will result in the acquisition of higher achievements and progress.

Last but not the least, it will be good for the practitioners to pay attention to make musical academic institutions more collaborative and innovative to motivate their students intrinsically and extrinsically which would further help to foster students’ music engagement. Once the students remain indulge with musical activities, his/her musical behavior and aesthetic experience will be influence, this further brings forth projection in their achievements.

## Limitations and future research

This research has certain limitations that should be considered in future studies. First, as the data were exclusively collected from China, the generalizability of the findings to other contexts may be limited. Subsequent research can examine the study model across diverse. The cross-sectional method used in this study doesn't dismiss the possibility of a long-term causal relationship arising from changes in student's motives, contentment, and musical preferences over time. Consequently, future research aiming to assess the validity of the study's model could use longitudinal data for a more comprehensive understanding. Second, musical engagement undoubtedly impacts students’ achievement and development, and the current research has only explored the mediating mechanism of music listening behavior and musical aesthetic experience in the music listening willingness, music engagement and musical achievement connection. Future studies should explore other areas of music domain, such as the association between music students in-school achievement with the progress and development in the music industry. Furthermore, the current study has examined the effect of general music engagement on students’ musical achievement, but future studies may investigate the effect of different types of music such as, cultural, rape, classical, etc. on students’ music achievement in different musical institutions. Finally, the data for this study has been collected from university students but the future researcher may collect data from diverse samples including teachers, parents and peers, and examine the association with the student’s musical progress and achievement ratio.

## Conclusion

The current study determines the influence of music listening willingness and musical engagement which may foster musical development and achievement of university music students in the light of self-determination theory (SDT). Specifically, the researchers examining the mediating role of musical aesthetic experience and listening music behavior in association with the music listening willingness, music engagement, and students’ musical achievement and development, particularly in Chinese universities. This study confirms the existence of a positive and significant link between music listening willingness and music engagement, as well as music listening behavior and aesthetic experience, which promotes students' musical achievement and development. Moreover, the researcher identified that there is a full mediation between listening music willingness and musical achievement and development through music listening behavior. Whereas, there is a partial mediation relationship between music engagement and musical achievement and development via music aesthetic experience. The outcomes of this study contribute and add to the music literature, particularly in the music educational domain, by evaluating the potential factors affecting students' musical achievement. This research provides significant empirical evidence to support the outcomes, and adds to both the theory and practice of musical education, particularly music education and its development in higher educational institutions (HEIs). Therefore, the findings of this research are likely to be considered valuable contributions in music literature, especially in the field of music education, and will help stakeholders and policymakers to understand the requirements of music education by highlighting the attributes of students’ motivational determinants. The study's authors are enthusiastic and hopeful that this research will provide novel directions for academia who have a strong desire to study university students’ musical activities and their positive consequences in the form of both academic and non-academic achievements.

## Data Availability

Correspondence and requests for materials should be addressed to X.W.

## Code availability

No custom code has been written for this dataset.
